# Primary antiphospholipid syndrome complicated by recurrent acute ST-elevation myocardial infarction: a case report

**DOI:** 10.3389/fcvm.2025.1656890

**Published:** 2026-01-07

**Authors:** Liming Huo, Chang Hou, Chengfu Cao, Xin Peng, Jian Liu

**Affiliations:** Department of Cardiology, Peking University People’s Hospital, Beijing, China

**Keywords:** acute ST-elevation myocardial infarction, antiphospholipid syndrome, coronary physiology, coronary microvascular dysfunction, immunomodulation

## Abstract

Clinical management of primary antiphospholipid syndrome (PAPS) complicated by acute myocardial infarction (AMI) is particularly challenging, especially when recurrent AMI and thrombocytopenia are present. This case report describes a 35-year-old male patient with PAPS who experienced two episodes of acute inferior ST-elevation myocardial infarction (STEMI). Comprehensive diagnostic evaluations including coronary imaging, physiology, immunology, bone marrow morphology, and molecular biology were performed to confirm the diagnosis. A personalized treatment regimen combining clopidogrel, low-molecular-weight heparin, methylprednisolone, hydroxychloroquine, and rituximab was administered, which resulted in a favorable long-term prognosis. This case underscores the importance of a multidisciplinary approach and individualized treatment strategies based on coronary functional assessment for managing patients with PAPS and AMI.

## Introduction

Primary antiphospholipid syndrome (PAPS) is a rare autoimmune condition characterized by thrombosis and/or pregnancy morbidity in the presence of persistent antiphospholipid antibodies (1). The complexity of this syndrome escalates when compounded with acute myocardial infarction (AMI), presenting significant diagnostic and therapeutic challenges due to its multifaceted pathophysiology involving both thrombotic and inflammatory pathways. This case report delves into the intricate management of a 35-year-old male diagnosed with PAPS who experienced recurrent episodes of acute inferior ST-elevation myocardial infarction (STEMI). Through comprehensive evaluations, including coronary imaging, physiology assessments, immunological profiling, bone marrow morphology, and molecular biology analysis, this report elucidates the critical importance of adopting a multidisciplinary approach and individualized treatment strategies. Furthermore, it highlights the necessity of coronary functional assessment for guiding diagnosis and therapy in patients with complex conditions such as PAPS complicated by AMI. By detailing the patient's journey from initial presentation through to long-term follow-up, we aim to contribute valuable insights into the optimal management of similar cases, underscoring the pivotal role of personalized medicine in achieving favorable outcomes.

## Case presentation

### Medical history

A 35-year-old male, presented with a 3-month history of skin petechiae and acute chest pain lasting 17 h. The patient had a history of psoriasis, fatty liver disease, and elevated transaminase levels. Laboratory tests revealed hyperuricemia (uric acid 548 μmol/L), mild dyslipidemia (triglycerides 1.75 mmol/L, LDL-C 2.13 mmol/L), and moderate hepatic steatosis on abdominal ultrasound, consistent with metabolic syndrome (2). He denied any history of connective tissue diseases, had a 15-year smoking history (10 cigarettes/day), and a family history of diabetes. Throughout the course of his psoriasis, he had not received systemic treatment, relying only on intermittent use of traditional Chinese medicine for symptomatic management.

Three months prior to admission, the patient had developed unprovoked petechiae on his limbs. Sixteen days before presentation, he visited an outpatient clinic due to oral blood blisters. Initial laboratory tests showed significant thrombocytopenia (2 × 10^9^/L), positive antinuclear antibodies (1:80), lupus anticoagulant (dRVVT-R: 1.34, normal 0.8–1.2), and anticardiolipin antibodies(31.5 U/mL, normal 0–10 U/mL). Following treatment with dexamethasone, intravenous immunoglobulin (IVIG), and intermittent platelet transfusions, his platelet count improved to 56 × 10^9^/L. Herombopag and prednisone were subsequently added to the treatment regimen. Seventeen hours prior to admission, he experienced persistent left-sided chest pain and tightness. Emergency evaluation revealed elevated high-sensitivity cardiac troponin I (hs-cTnI 1,011 pg/mL, normal <17.8 pg/mL), and ST-segment elevation in leads III and aVF on electrocardiogram. An initial diagnosis of acute inferior STEMI was made, and the patient was admitted to the cardiac care unit after receiving aspirin and clopidogrel as antiplatelet therapy. Atorvastatin 10 mg once daily at bedtime was initiated during hospitalization.

### Physical examination

On admission, the patient's temperature was 36.5 °C, pulse rate was 84 beats/min, respiratory rate was 17 breaths/min, and blood pressure was 117/87 mmHg. He was 1.75 m tall and weighed 75 kg, yielding a body mass index (BMI) of 24.65 kg/m² (3). There were no rashes on his face, but scattered areas of depigmentation were noted on the right ear, neck, back, and limbs. Petechiae or ecchymoses were not observed. Cardiac, pulmonary, and abdominal examinations were unremarkable, and no joint swelling or tenderness was observed.

### Investigations

**Figure 1:**
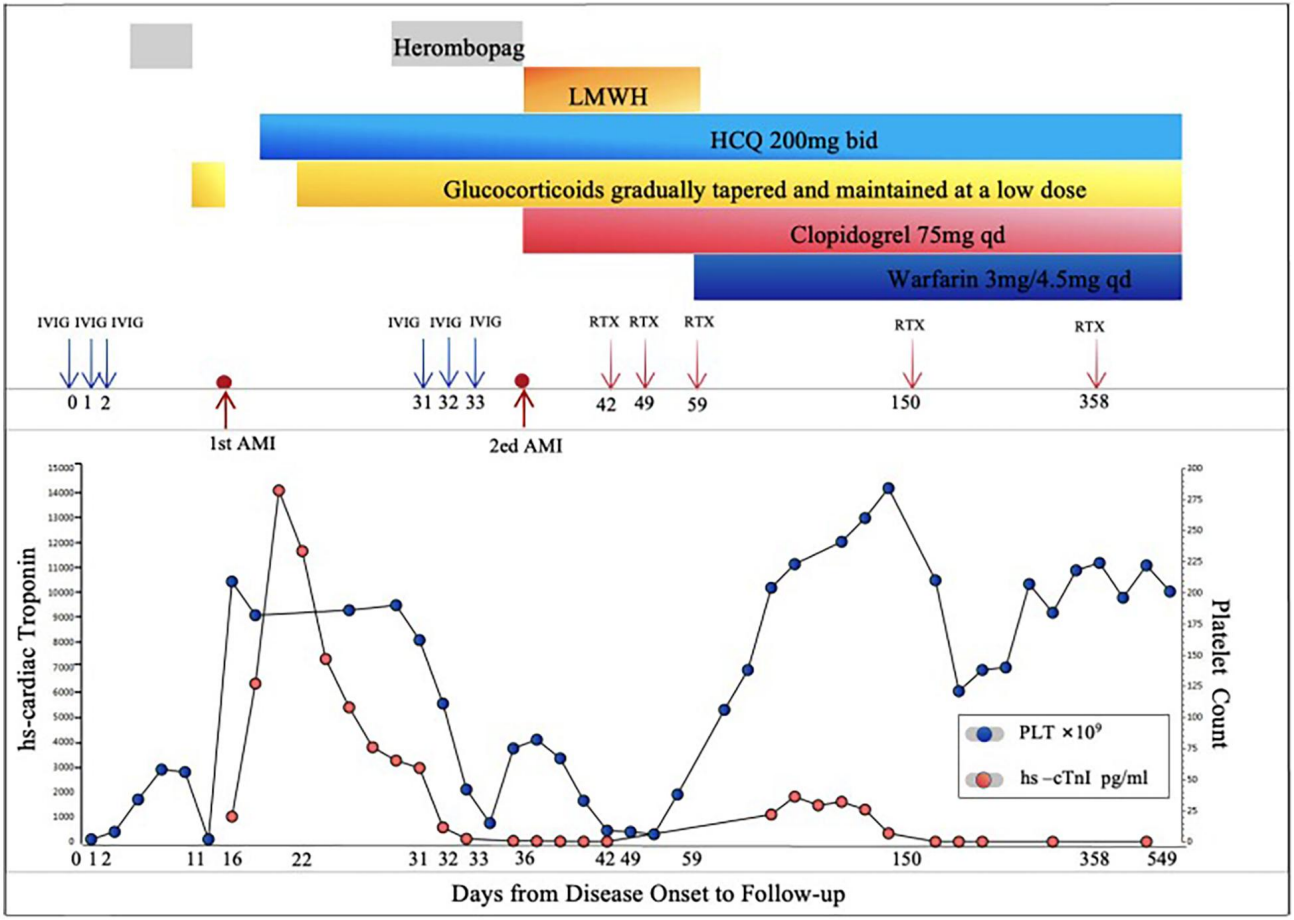
Clinical course and laboratory findings of a patient during treatment and follow-up. This figure illustrates the treatment course and laboratory findings of the patient from disease onset to follow-up. The horizontal axis represents days from onset to follow-up, the left vertical axis shows high-sensitivity cardiac troponin (high-sensitivity cardiac troponin I, Hs-cTnI) levels, and the right vertical axis indicates platelet count. During the treatment period, the patient received intravenous immunoglobulin (Intravenous Immunoglobulin, IVIG) at a dose of 20 g on days 0, 1, 2, and again on days 31, 32, and 33. Rituximab (Rituximab, RTX) was initially administered at a dose of 200 mg on day 36, followed by 500 mg on days 42, 49, 57,150, and 358. From day 11 onward, the patient orally took Herombopag at a dose of 7.5 mg to increase platelet count. Additional treatments included low-molecular-weight heparin (low-molecular-weight heparin, LMWH) from day 16 to day 59, hydroxychloroquine (hydroxychloroquine, HCQ) starting on day 19 at 200 mg twice daily (bid), glucocorticoids (glucocorticoids, GC) initiated on day 22 with gradual tapering and maintenance at low doses, clopidogrel (clopidogrel, CLP) started on day 36 at 75 mg once daily (qd), and warfarin (Warfarin, WFR) initiated on day 59 at 3 mg/4.5 mg qd. High-sensitivity cardiac troponin levels peaked on days 17 and 37, indicating two episodes of acute myocardial infarction (acute myocardial infarction, AMI), thereafter gradually returning to normal. Platelet counts progressively increased and stabilized over time. Overall, the treatment regimen effectively improved clinical parameters and promoted disease stabilization and recovery.

**Figure 2:**
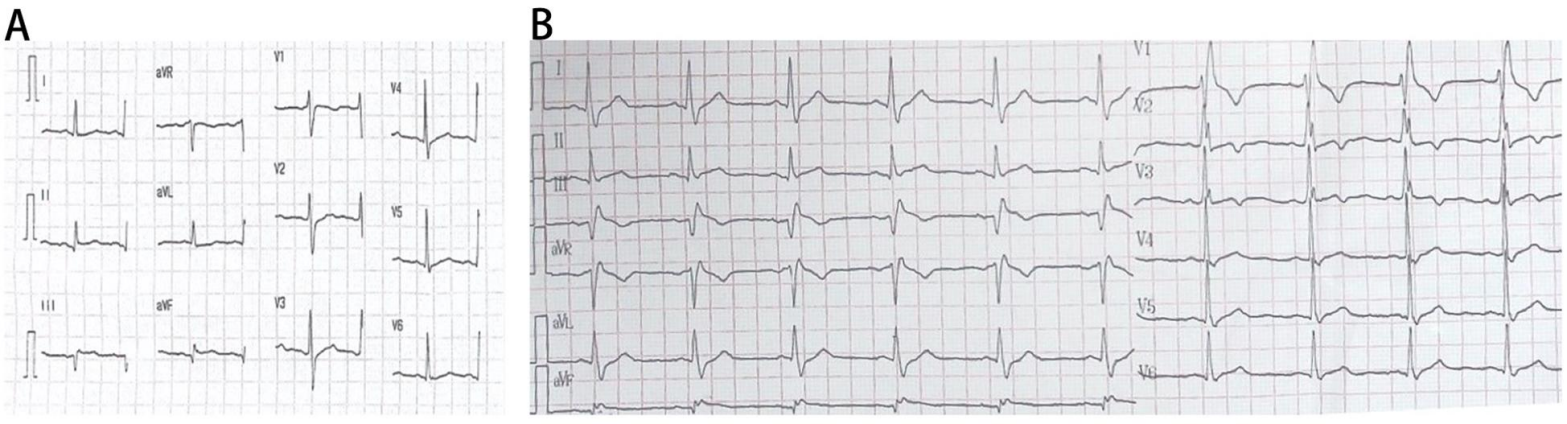
Serial electrocardiographic (ECG) findings during hospitalization and recurrent chest pain. **(A)** Resting ECG during hospitalization: the resting electrocardiogram (ECG) obtained during hospitalization demonstrates sinus rhythm with no axis deviation. Pathological Q waves are observed in leads II, III, and aVF, suggesting possible inferior wall myocardial infarction. **(B)** ECG during recurrent chest pain: the ECG recorded during an episode of recurrent chest pain shows sinus rhythm with no axis deviation. Persistent pathological Q waves are present in leads II, III, and aVF. Additionally, there is slight ST-segment elevation in lead III and a new-onset right bundle branch block (RBBB), indicating further cardiac complications.

**Figure 3:**
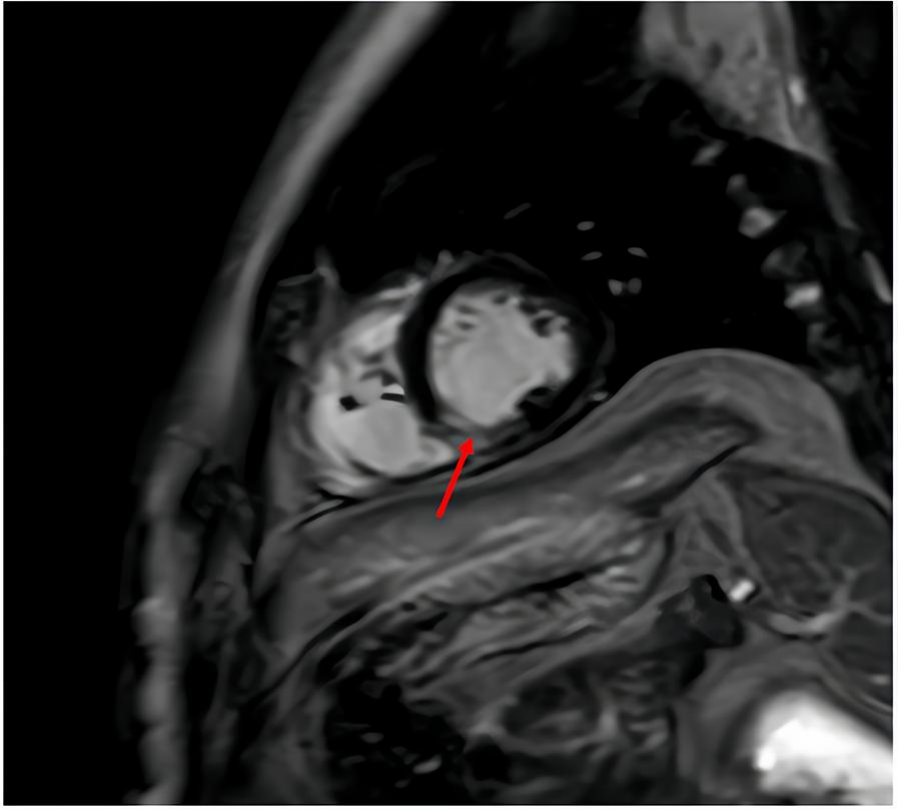
Cardiac magnetic resonance imaging (MRI) with contrast enhancement obtained during hospitalization. The arrow indicates transmural delayed enhancement in the mid-to-distal inferoseptal and mid-inferior segments of the left ventricle, which is indicative of myocardial infarction.

**Figure 4:**
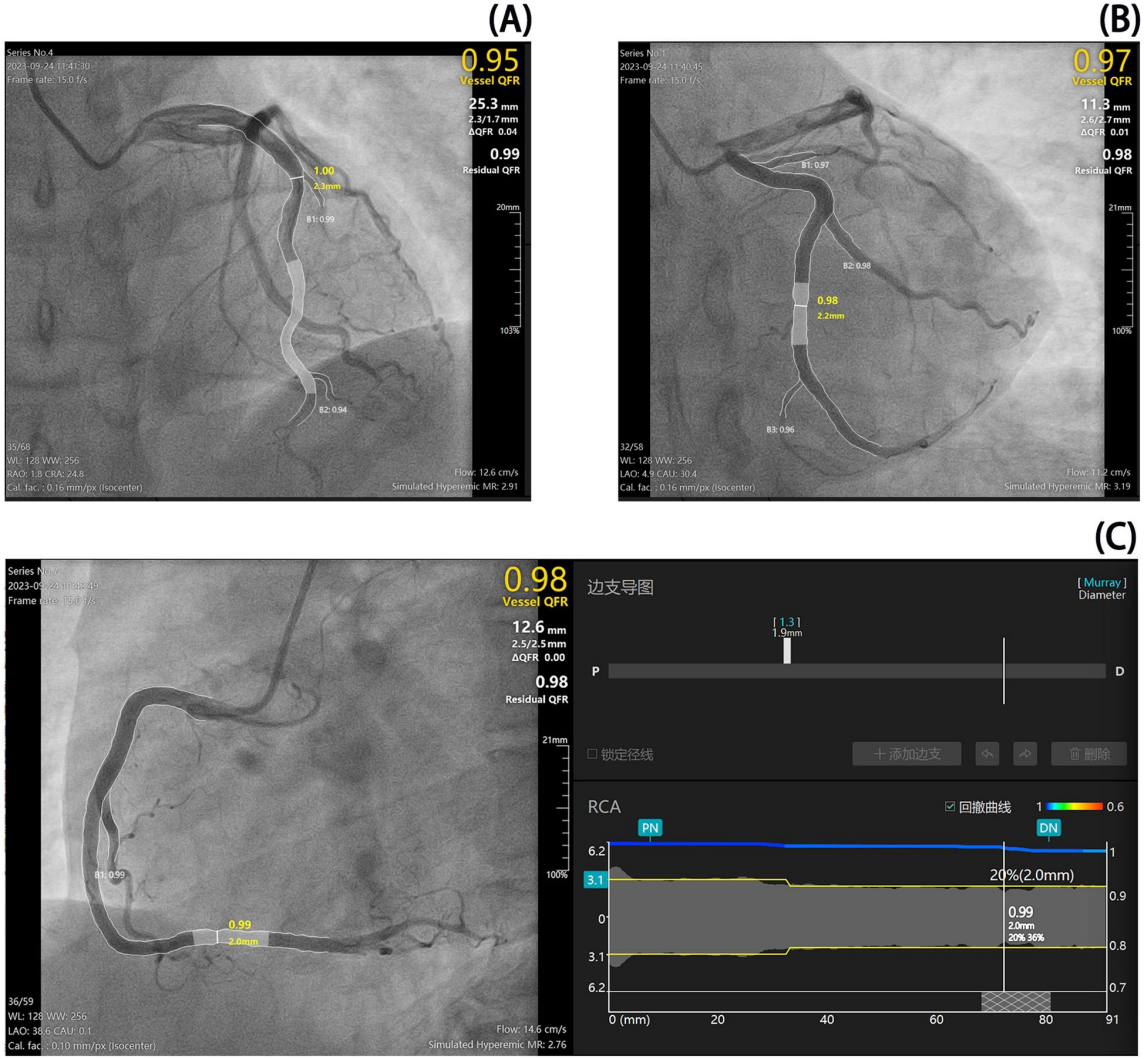
Coronary angiography and microvascular resistance measurements. Coronary angiography revealed no significant stenosis in the major epicardial vessels. Quantitative flow ratio (QFR) measurements were within normal limits for the left anterior descending artery (LAD, QFR 0.95), left circumflex artery (LCX, QFR 0.97), and right coronary artery (RCA, QFR 0.98). However, angiographic microvascular resistance (AMR) measurements were elevated across all vessels, with values of 291 for LAD, 319 for LCX, and 276 for RCA. An AMR value ≥266 mmHg s/m is indicative of coronary microcirculatory dysfunction, suggesting that this patient exhibits diffuse microvascular impairment despite the absence of obstructive epicardial disease. The shaded (gray) regions in the panels indicate the target segments used for QFR and AMR measurements.

On admission, the platelet count was normal (182 × 10^9^/L), transaminase levels were elevated (ALT 96 U/L, normal 9–50 U/L;AST 41 U/L, normal 15–40 U/L), and uric acid levels were high (548 μmol/L, normal 208–428 μmol/L). Autoimmune profiling confirmed antiphospholipid syndrome, with persistent positivity for antinuclear antibodies, lupus anticoagulant, and IgG anticardiolipin antibodies (Supplementary Table S1). Hematologic assessment revealed no evidence of malignancy, showing grade III bone marrow hyperplasia, adequate nucleated cells, impaired megakaryocyte maturation, and reduced platelet production; immunophenotyping, cytogenetics, and molecular studies were unremarkable. Infection-related tests were positive for SARS-CoV-2 RNA, while other respiratory viruses, enteroviruses, blood cultures, and atypical pathogens were negative. Urinalysis revealed proteinuria (24-h urine protein: 140 mg). Acute inferior STEMI was confirmed by elevated troponin levels (hs-cTnI peak 11,673.4 pg/mL) (Figure 1) and ECG findings (pathological Q waves in leads III and aVF) (Figure 2A). Cardiac MRI with contrast enhancement showed delayed enhancement in the mid-to-distal inferoseptal and mid-inferior segments of the left ventricle (Figure 3). Coronary angiography showed no significant stenosis in the major epicardial vessels, and quantitative flow ratio (QFR) values were normal (LAD: 0.95; LCX: 0.97; RCA: 0.98), but angiographic microvascular resistance (AMR) was elevated in all three major vessels (LAD: 291; LCX: 319; RCA: 276; cutoff ≥266 mmHg s/m), indicating coronary microvascular dysfunction (4) (Figure 4).

### Treatment and management

Coronary angiography did not show significant epicardial vessel stenosis or thrombus formation; however, coronary microcirculatory dysfunction was evident (Figure 4). Based on ECG and cardiac injury marker dynamics as well as delayed enhancement features on cardiac MRI, the patient was diagnosed with acute inferior STEMI. Further laboratory tests ruled out connective tissue diseases, infectious diseases, and hematological malignancies. Antiphospholipid antibodies, including lupus anticoagulant and IgG anticardiolipin antibodies, were confirmed positive on two separate occasions more than 12 weeks apart (with aCL levels of 31.5 U/mL, 63.7 U/mL, and 54.9 U/mL in sequential tests), fulfilling the 2006 Sydney criteria for definite antiphospholipid syndrome (APS) (5, 6). Anti-β₂-glycoprotein I antibodies were negative. These findings led to a final diagnosis of PAPS.

Initial therapy included clopidogrel, low-molecular-weight heparin(LMWH), and hydroxychloroquine as antithrombotic and immunomodulatory therapies. The patient's condition improved, but his platelet count continued to decline (lowest 8 × 10^9^/L during hospitalization). Antithrombotic drugs were discontinued to prevent bleeding events and glucocorticoids and herombopag were resumed. However, the patient experienced recurrent chest pain and dynamic changes in cardiac injury markers and ECG findings (Figure 2B), suggesting another MI (the patient refused repeat coronary angiography). After multidisciplinary consultation involving cardiology, rheumatology, hematology, and pharmacy, the diagnosis of PAPS complicated by AMI was confirmed. Given an adjusted Global Antiphospholipid Syndrome Score (aGAPSS) of 11, indicating high thrombotic risk, a comprehensive risk-benefit analysis was performed (7).

Notably, herombopag, a thrombopoietin receptor agonist, has been associated with increased platelet activation and prothrombotic risk in autoimmune thrombocytopenia, particularly in patients with underlying antiphospholipid antibodies (8). Its potential contribution to recurrent thrombosis in this context warrants clinical caution. The treatment plan included methylprednisolone (40 mg daily) followed by prednisone (40 mg daily), rituximab (initial dose 200 mg, then 500 mg × 3 doses), enoxaparin followed by warfarin (4.5 mg daily), and clopidogrel (75 mg daily), along with hepatoprotective and infection prophylaxis. The patient's chest pain resolved and platelet count stabilized prior to discharge (Figure 1).

### Follow-up

After discharge, the patient was regularly followed up by the cardiology and rheumatology departments. The patient did not experience recurrent chest pain, chest tightness, or bleeding from the skin, mucous membranes, or internal organs. Four months after discharge, his immune indicators improved, and he received a fourth dose of rituximab (500 mg). Prednisone and hydroxychloroquine were continued to treat the underlying diseases. Six months post-discharge, his immune indicators remained stable. One year after discharge, cardiac injury markers and antiphospholipid antibodies were negative, and echocardiography showed no significant blood flow abnormalities. He received another rituximab infusion (500 mg) and continued prednisone (5 mg/7.5 mg every other day) and hydroxychloroquine (200 mg twice daily). Antithrombotic therapy with clopidogrel (75 mg daily) and warfarin (4.5 mg daily) was maintained along with treatments for osteoporosis prevention and lipid stabilization (Figure 1).

## Discussion and conclusion

APS is an autoimmune disorder characterized by the presence of antiphospholipid antibodies, leading to recurrent venous and arterial thrombosis, thrombocytopenia, and elevated antiphospholipid antibody titers. It may be classified as primary or secondary (9). Vascular involvement can manifest as venous thromboembolism, arterial thrombosis, or microvascular dysfunction. Cardiac involvement can result in acute MI, ischemic cardiomyopathy, microcirculatory dysfunction, and valvular damage. Without appropriate intervention, patients face substantial risks of recurrent thrombosis or hemorrhage, with reported recurrence rates up to 50% (7, 10, 11).

Previous studies have identified causes of MI in patients with APS, including acute thrombosis and coronary spasm (10, 12). In this case, the patient experienced recurrent chest pain and dynamic changes in ECG and cardiac injury marker levels during treatment with glucocorticoids and herombopag. Coronary angiography, QFR, and AMR findings suggested coronary microvascular dysfunction, raising the possibility of coronary spasm or thrombosis. Conservative antithrombotic therapy was initiated, highlighting the unique value of coronary physiological indices for diagnosing and guiding treatment in AMI (13). However, optical coherence tomography (OCT) or intravascular ultrasound (IVUS) were not performed to confirm the presence of plaque rupture. The patient's unique feature was the occurrence of recurrent MI despite treatment for PAPS, which may be related to the thrombotic adverse effects of herombopag and the thrombotic tendency of the underlying disease (14).

Given the dual risk of thrombosis and bleeding, individualized anticoagulation and immunomodulation are essential. The common anticoagulants include heparin, unfractionated heparin, and warfarin. In cases of severe thrombocytopenia, glucocorticoids and immunosuppressants can be used (9). In this patient, the addition of rituximab prevented further bleeding and chest pain, and his platelet count gradually recovered, with negative antiphospholipid antibodies.

The use of rituximab in APS remains off-label, and optimal dosing is not standardized. Regimens vary across reports, including 375 mg/m² weekly for four weeks, low-dose protocols (e.g., single 500 mg or 1,000 mg), or split high-dose infusions (15). Emerging evidence suggests that both standard- and low-dose rituximab can achieve significant reductions in anticardiolipin antibody titers and clinical improvement, likely through depletion of autoreactive B cells (15). Given the lower B-cell burden in autoimmune diseases compared to lymphomas and the risk of infusion reactions, individualized dosing based on disease severity, organ involvement, and comorbidities may offer a favorable risk-benefit profile.

Notably, one year after discharge, the patient achieved seronegativity for antiphospholipid antibodies, potentially reflecting sustained B-cell suppression by rituximab. However, coronary microvascular dysfunction persisted as a challenging complication, difficult to monitor and treat. While cardiac MRI and quantitative coronary physiology (e.g., AMR) can detect microvascular impairment (16), targeted therapies for microvascular injury in APS remain lacking. This highlights the need for further research into both pathophysiology and effective interventions for microvascular disease in this population.

In summary, patients with PAPS complicated by AMI should consider using coronary functional or intravascular imaging to guide the diagnosis and treatment. A comprehensive assessment of thrombotic and bleeding risks, along with individualized immunomodulatory and antithrombotic strategies, is essential for improving the long-term outcomes.

## Data Availability

The original contributions presented in the study are included in the article/Supplementary Material, further inquiries can be directed to the corresponding author.

## Supplementary material

Supplementary Table S1 Autoimmune Profile for Antipholipid Syndrome (APS) Patients. Supplementary Table S1 provides a comprehensive autoimmune profile of patients diagnosed with Antiphospholipid Syndrome (APS), including measurements of various autoantibodies, immunoglobulins, complement components, and coagulation factors. Key findings include elevated levels of Anti-Cardiolipin Antibody (aCL) at 31.5 U/mL, an increased Standardized Dilute Russell's Viper Venom Time (dRVVT) Ratio of 1.34, elevated Immunoglobulin G (IgG) at 19.95 g/L, reduced Immunoglobulin M (IgM) at 0.34 g/L, and decreased Complement Component C1q at 120 mg/L, all of which support the diagnosis of APS and suggest underlying immune dysregulation. Additional testing for anti-double-stranded DNA (anti-dsDNA) antibodies, anti-β2-glycoprotein I antibodies, anti-nuclear antibodies (ANA), extractable nuclear antigen (ENA) antibodies, as well as coagulation parameters such as Antithrombin Activity, Protein C Activity, and Protein S Activity, were also performed to evaluate disease activity and thrombotic risk. These results emphasize the importance of a systematic and comprehensive approach to autoimmune profiling in the clinical assessment and long-term management of patients with APS.

## References

[1] Murphy P. Thrombocytopenia and antiphospholipid syndrome. Cardiovasc Drugs Ther. (2025). 10.1007/s10557-025-07730-0 40478491

[2] Hamooya BM Siame L Muchaili L Masenga SK Kirabo A. Metabolic syndrome: epidemiology, mechanisms, and current therapeutic approaches. Front Nutr. (2025) 12:1661603. 10.3389/fnut.2025.1661603 40969606 PMC12441046

[3] Rubino F Cummings DE Eckel RH Cohen RV Wilding JPH Brown WA Definition and diagnostic criteria of clinical obesity. Lancet Diabetes Endocrinol. (2025) 13(3):221–62. 10.1016/s2213-8587(24)00316-4 39824205 PMC11870235

[4] Gao B Wu G Xie J Ruan J Xu P Qian Y Quantitative flow ratio-derived index of microcirculatory resistance as a novel tool to identify microcirculatory function in patients with ischemia and no obstructive coronary artery disease. Cardiology. (2024) 149(1):14–22. 10.1159/000534287 37839404 PMC10836850

[5] Zhu QN Qi XB Ren SW Li YY Yan ZW Sun Y Wang: novel advances on pathophysiological mechanisms, clinical manifestations, and treatment of antiphospholipid syndrome. Front Immunol. (2025) 16:1639065. 10.3389/fimmu.2025.1639065 40918089 PMC12408547

[6] Kaul M Erkan D Sammaritano L Lockshin DM. Assessment of the 2006 revised antiphospholipid syndrome classification criteria. Ann Rheum Dis. (2007) 66(7):927–30. 10.1136/ard.2006.067314 17337473 PMC2497429

[7] Radin M Schreiber K Costanzo P Cecchi I Roccatello D Baldovino S The adjusted global AntiphosPholipid syndrome score (aGAPSS) for risk stratification in young APS patients with acute myocardial infarction. Int J Cardiol. (2017) 240:72–7. 10.1016/j.ijcard.2017.02.155 28385357

[8] Gonzalez-Porras JR Bastida MJ. Eltrombopag in immune thrombocytopenia: efficacy review and update on drug safety. Ther Adv Drug Saf. (2018) 9(6):263–85. 10.1177/2042098618769587 29854389 PMC5971401

[9] Sammaritano LR. Antiphospholipid syndrome. Best Pract Res Clin Rheumatol. (2020) 34(1):101463. 10.1016/j.berh.2019.101463 31866276

[10] Gandhi H Ahmed N Spevack MD. Prevalence of myocardial infarction with non-obstructive coronary arteries (MINOCA) amongst acute coronary syndrome in patients with antiphospholipid syndrome. Int J Cardiol Heart Vasc. (2019) 22:148–9. 10.1016/j.ijcha.2018.12.015 30766913 PMC6360345

[11] Gan Y Zhao Y Li G Ye H Zhou Y Hou C Risk factors and outcomes of acute myocardial infarction in a cohort of antiphospholipid syndrome. Front Cardiovasc Med. (2022) 9:871011. 10.3389/fcvm.2022.871011 35865377 PMC9294316

[12] Correia AF Oliveira DC Sanctos M. Coronary artery thromboses, stent thrombosis and antiphospholipid antibody syndrome: case report. Cardiol Res. (2018) 9(2):129–32. 10.14740/cr661w 29755633 PMC5942245

[13] Naoum I Schliamser JE Zissman K Fuks A Zafrir B. Multimodality imaging in a young patient with antiphospholipid syndrome-related acute myocardial infarction. Eur Heart J Cardiovasc Imaging. (2024) 25(9):e205. 10.1093/ehjci/jeae088 38547458

[14] Gunes H Kivrak T. Eltrombopag induced thrombosis: a case with acute myocardial infarction. Curr Drug Saf. (2016) 11(2):174–6. 10.2174/1574886311207040255 26560493

[15] Gan Y Zhong X Zhao Y Li G Ye H Li C. Low dose versus standard dose rituximab for the treatment of antiphospholipid syndrome: a pilot study from a tertiary medical center. Front Immunol. (2022) 13:971366. 10.3389/fimmu.2022.971366 36405743 PMC9670802

[16] Chen W Ni M Huang H Cong H Fu X Gao W Chinese expert consensus on the diagnosis and treatment of coronary microvascular diseases (2023 edition). MedComm (2020). (2023) 4(6):e438. 10.1002/mco2.438 38116064 PMC10729292

