# Supplementary material for: Primary antiphospholipid syndrome complicated by recurrent acute ST-elevation myocardial infarction: a case report
Source: Front Cardiovasc Med. 2026 Jan 7;12:1656890. doi: 10.3389/fcvm.2025.1656890 (PMC12819726; doi:10.3389/fcvm.2025.1656890)
Supplement: Supplementary Table S1 — Autoimmune Profile for Antipholipid Syndrome (APS) Patients. Supplementary Table S1 provides a comprehensive autoimmune profile of patients diagnosed with Antiphospholipid Syndrome (APS), including measurements of various autoantibodies, immunoglobulins, complement components, and coagulation factors. Key findings include elevated levels of Anti-Cardiolipin Antibody (aCL) at 31.5 U/mL, an increased Standardized Dilute Russell's Viper Venom Time (dRVVT) Ratio of 1.34, elevated Immunoglobulin G (IgG) at 19.95 g/L, reduced Immunoglobulin M (IgM) at 0.34 g/L, and decreased Complement Component C1q at 120 mg/L, all of which support the diagnosis of APS and suggest underlying immune dysregulation. Additional testing for anti-double-stranded DNA (anti-dsDNA) antibodies, anti-β2-glycoprotein I antibodies, anti-nuclear antibodies (ANA), extractable nuclear antigen (ENA) antibodies, as well as coagulation parameters such as Antithrombin Activity, Protein C Activity, and Protein S Activity, were also performed to evaluate disease activity and thrombotic risk. These results emphasize the importance of a systematic and comprehensive approach to autoimmune profiling in the clinical assessment and long-term management of patients with APS. [file Table1.docx]

**Table S1** Autoimmune Profile for Antipholipid Syndrome (APS) Patients

| No. | Item Name | Result | Qualitative | Reference Value | Unit |
| --- | --- | --- | --- | --- | --- |
| 1 | Anti-Nuclear Antibodies (ANA) | 1:80 Speckled |  | <1:40 | Semi-quantitative |
| 2 | Anti-ENA Antibody - Sm | 2.22 |  | 0-20 | RU/mL |
| 3 | Anti-ENA Antibody - U1RNP | 1.88 |  | 0-20 | RU/mL |
| 4 | Anti-ENA Antibody - SSA (60kDa) | 12.26 |  | 0-20 | RU/mL |
| 5 | Anti-ENA Antibody - SSB | 1.11 |  | 0-20 | RU/mL |
| 6 | Anti-Jo-1 Antibody | 0.51 |  | 0-20 | RU/mL |
| 7 | Mitochondrial Antibody IgG M2 | 15.57 |  | 0.00-30.00 | RU/mL |
| 8 | Anti-Thyroid Peroxidase Antibody | 0.94 |  | 0.00-20.00 | RU/mL |
| 9 | Anti-Proteinase 3 Antibody | 1.05 |  | 0.00-20.00 | RU/mL |
| 10 | Anti-Endothelial Cell Antibody | Negative |  | Negative |  |
| 11 | Anti-Scl-70 Antibody | <0.50 |  | 0-20 | RU/mL |
| 12 | Ro-52 | 11.21 |  | 0-20 |  |
| 13 | Anti-Double-Stranded DNA Antibody (Anti-dsDNA) | 12.4 |  | 0-25.0 | IU/mL |
| 14 | Anti-Glycoprotein Antibody | 1.84 |  | 0.00 - 20.00 | RU/mL |
| 15 | Anti-Cardiolipin Antibody | 31.5 | ↑ | 0-10.0 | U/mL |
| 16 | Anti-β2-Glycoprotein I Antibody | 16.21 |  | 0.00 - 20.00 | RU/mL |
| 17 | dRVVT Screening Test | 48.9 |  | None | s |
| 18 | dRVVT Confirmation Test | 30.4 |  | None | s |
| 19 | Standardized dRVVT Ratio | 1.34 | ↑ | 0.8 - 1.2 |  |
| 20 | Antithrombin Activity | 114 |  | 83 - 128 | % |
| 21 | Protein C Activity | 136 |  | 70 - 140 | % |
| 22 | Protein S Activity | 91.1 |  | 63.5 - 149 | % |
| 23 | Immunoglobulin G (IgG) | 19.95 | ↑ | 7.00 - 16.00 | g/L |
| 24 | Immunoglobulin A (IgA) | 2.67 |  | 0.70 - 4.00 | g/L |
| 25 | Immunoglobulin M (IgM) | 0.34 | ↓ | 0.40 - 2.30 | g/L |
| 26 | Complement C3 | 1.38 |  | 0.90 - 1.80 | g/L |
| 27 | Complement C4 | 0.39 |  | 0.10 - 0.40 | g/L |
| 28 | Complement C1q | 120 | ↓ | 159 - 233 | mg/L |
| 29 | Anti-Streptolysin O | < 100.0 |  | 0.0 - 200.0 | IU/mL |
| 30 | Rheumatoid Factor | < 5.0 |  | 0.0 - 14.0 | IU/mL |
